# Sex-Specific Relationships between HDL-Cholesterol Levels and 10-Year Mortality in Individuals with Atherosclerotic Cardiovascular Disease: A Nationwide Cohort Study of South Koreans

**DOI:** 10.3390/metabo13121175

**Published:** 2023-11-26

**Authors:** Hyun Suk Yang, Ho Jin Jeong, Hyeongsu Kim, Seungho Lee, Mina Hur

**Affiliations:** 1 Departments of Cardiovascular Medicine, Research Institute of Medical Science, Konkuk University School of Medicine, Seoul 05029, Republic of Korea; yang.hyun@kuh.ac.kr; 2 Department of Preventive Medicine, Konkuk University School of Medicine, Seoul 05029, Republic of Korea; wyntme00@naver.com (H.J.J.); mubul@kku.ac.kr (H.K.); 3 Departments of Preventive Medicine, Dong-A University School of Medicine, Busan 49315, Republic of Korea; seunglee@dau.ac.kr; 4 Department of Laboratory Medicine, Research Institute of Medical Science, Konkuk University School of Medicine, Seoul 05029, Republic of Korea

**Keywords:** high-density lipoprotein cholesterol, atherosclerotic cardiovascular diseases, all-cause mortality, biomarker

## Abstract

Large epidemiological studies show U-shaped relationships between high-density lipoprotein cholesterol (HDL-C) levels and all-cause mortality in individuals without atherosclerotic cardiovascular diseases (ASCVD). Association in those with ASCVD by sex is unclear. We examined the association between HDL-C levels and 10-year all-cause mortality in subjects (≥40 years of age) with ASCVD using the 2010 National Health Insurance Service and the National Death Registry of Korea. We categorized HDL-C levels into three groups (low: <40 mg/dL for males, <50 mg/dL for females; high: 40–90 mg/dL for males, 50–90 mg/dL for females; extremely high: >90 mg/dL) and 10 mg/dL intervals. We conducted a sex-stratified and adjusted Cox proportional hazards analysis. Out of 1,711,548 individuals (54% female, mean age 61.4 years), 10-year mortality was observed in 218,252 (12.8%). Males had a higher mortality rate than females (16.2% vs. 9.8%; *p* < 0.001). When adjusting for age, body mass index, LDL-cholesterol, triglycerides, hypertension, diabetes, smoking, and alcohol consumption, the low and extremely high HDL-C groups had significantly higher hazard ratios for 10-year mortality compared to the high HDL-C group in males [1.183 (1.166–1.199), 1.359 (1.288–1.434)] and in females [1.153 (1.138–1.169), 1.095 (1.029–1.167)]. The frequency distribution bars for the 10-year mortality rate showed sex-specific nadirs of 50–59 mg/dL in males and 70–79 mg/dL in females. In this ASCVD cohort, the extremely high HDL-C (>90 mg/dL) group had 35.9% and 9.5% higher 10-year mortality risks than the high HDL-C group for males and females, respectively. There was a slightly U-shaped relationship between baseline HDL-C levels and a 10-year mortality rate, with earlier inflection in males than in females.

## 1. Introduction

In epidemiological studies of the general population, those with atherosclerotic cardiovascular disease (ASCVD) are often excluded. It has been observed that the level of high-density lipoprotein cholesterol (HDL-C) has a U-shaped or J-shaped association with all-cause mortality [1,2,3]. Various population characteristics, such as ethnicity, lifestyle, and comorbidities, lead to varying optimal HDL-C levels by sex in different studies. The CANHEART study [1] of individuals without pre-existing cardiovascular (CV) disease (N = 631,762, mean age 57.2 years) found that higher HDL-C levels (>70 mg/dL in men, >90 mg/dL in women) were associated with an increased risk of non-CV mortality. The Copenhagen study [2] with a general population (N = 116,508, median age 57.5 years) exhibited the lowest all-cause mortality risk in 73 mg/dL (95% CI: 54–77) in men and 93 mg/dL (95% CI: 69–97) in women. One of the Korean studies (N = 15,860,253, mean age 47.4 years) [3], which utilized the Korean National Health Insurance Service (NHIS) big data, reported that the lowest all-cause mortality was observed for an HDL-C range of 40–59 mg/dL in men and 50–79 mg/dL in women, after excluding pre-existing heart disease, stroke, cancer, or other diseases.

In South Korea, a biennial health check-up is mandatory and free of charge. Starting in 2009, the Korean NHIS began measuring HDL-C levels every 4 years in males aged 24 or older and females aged 40 or older, allowing for tracking of HDL-C-correlated all-cause mortality over a 10-year period. Our group recently published a paper [4] using the Korean NHIS database to assess a clinically ASCVD-free cohort (N = 5,703,897, mean age 52.6 years) to show a sex-specific U-shaped relationship between HDL-C levels and 10-year composite end-points of major adverse CV events (including all-cause mortality), emphasizing sex-specific HDL-C references for males (optimal: 40–70 mg/dL, extremely high: >90 mg/dL) and females (optimal: 50–110 mg/dL, extremely high: >130 mg/dL).

However, the association between HDL-C levels and all-cause mortality has not been extensively studied in populations with ASCVD, with no such studies in Korea. HDL-C levels in subjects with ASCVD vary greatly, while low-density lipoprotein cholesterol (LDL-C) levels are suppressed mostly via statins.

We tested the hypothesis that, in the population with ASCVD, extremely high HDL-C levels (>90 mg/dL) increase all-cause mortality in each sex, exploring the sex-specific relationship between baseline HDL-C levels and 10-year all-cause mortality.

## 2. Materials and Methods

### 2.1. Data Sources

The data for this study were obtained from two sources. The Korean NHIS provided medical and laboratory data related to healthcare in South Korea, while the National Death Registry of Korea provided information about all-cause mortality. The study protocol was designed in accordance with the Declaration of Helsinki criteria. It was approved by the Institutional Review Board of Konkuk University Medical Center (KUMC 2022-03-021) in Seoul, Korea, and the NHIS official review committee (NHIS-2023-1-237).

### 2.2. Study Design and Subject Selection

**Figure 1:**
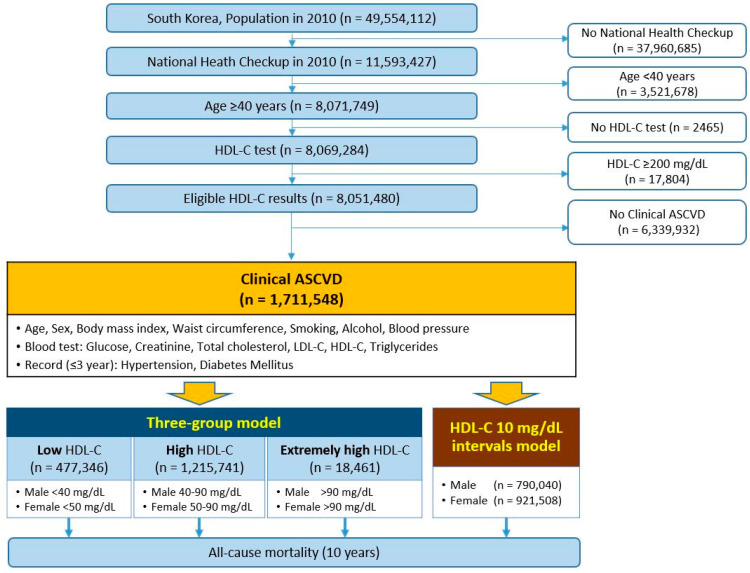
Flow diagram of the retrospective nationwide cohort study. Abbreviations: HDL-C, high-density lipoprotein cholesterol; LDL-C, low-density lipoprotein cholesterol; ASCVD, atherosclerotic cardiovascular diseases.

This study was a nationwide population-based cohort retrospective analysis. Figure 1 displays the study flowchart. In 2010, individuals aged 40 and above with eligible HDL-C results and clinical ASCVD were included, while those without HDL-C testing or HDL-C levels ≥ 200 mg/dL were excluded. To identify cases of clinical ASCVD, we utilized the eighth Korean Standard Classification of Diseases (KCD) codes. We defined clinical ASCVD as any one of the following codes found within three years prior to the index year: angina pectoris (I20), acute coronary syndrome (I21-24), chronic ischemic heart disease (I25), presence of a coronary angioplasty implant and graft (Z95.5), presence of aortocoronary bypass grafts (Z95.1), stroke (I63-66), transient ischemic attack (G45), or peripheral arterial disease (I73). In South Korea, patients diagnosed with ASCVD are prescribed drug therapy (generally statins) regardless of LDL-C levels. We did not include this as a variable because the individual details (brand, dosage, continuity, compliance, and effectiveness) would be extremely complicated.

### 2.3. Definitions and Models

Each participant’s index date was set as the day they underwent their national health checkup in 2010. On this date, information was collected regarding their age, sex, body mass index (BMI), waist circumference, frequency of smoking and alcohol consumption, blood pressure (BP), and blood test results. Blood samples were taken after an overnight fast and analyzed for glucose, serum creatinine, total cholesterol (TC), HDL-C, and triglycerides using standard laboratory methods. LDL-C was calculated using the Friedewald formula: LDL-C = TC − (triglycerides/5) − HDL-C. Direct LDL-C assays were not taken as they are not routinely offered in national health checkups [5]. Non-HDL cholesterol (Non-HDL-C) was calculated by subtracting HDL-C from TC. Obesity was defined as having a BMI greater than 25 kg/m^2^, while abdominal obesity was defined as a waist circumference equal to or greater than 90 cm for males and 85 cm for females [6]. Hypertension was defined by having a systolic BP of 140 mm Hg or higher on the index date or a hypertension-related eighth KCD code (I10*, I11*, I12*, I13*, I15*, I674) within three years of the index year. Diabetes mellitus was defined as having a fasting glucose level of 126 mg/dL or higher on the index date or a diabetes-related eighth KCD code (E10*, E11*) within three years of the index year.

Two models were employed to classify the levels of HDL-C (Figure 1). The first model was the sex-stratified three-group model, which classifies males with less than 40 mg/dL and females with less than 50 mg/dL as the ‘low group’, while males with 40–90 mg/dL and females with 50–90 mg/dL are in the ‘high group’, and those with more than 90 mg/dL are in the ‘extremely high group’. This model was based on two guidelines: (1) the 2019 ACC/AHA guidelines on primary prevention of CV disease, which consider low HDL-C levels as a risk factor for men at <40 mg/dL and women at <50 mg/dL [7], and (2) the 2019 ESC/EAS guidelines for the management of dyslipidemia, which state that extremely high HDL-C levels (>90 mg/dL) may increase the risk of ASCVD [8]. The second model involved creating 10 mg/dL intervals for the baseline levels of HDL-C. All subjects were monitored for ten years to track all-cause mortality. Survival time was calculated from the index date to the date of death.

### 2.4. Statistical Analysis

Data were presented as frequency (percentage) for categorical variables and mean ± standard deviation or median and interquartile range for continuous variables. We compared baseline characteristics between males and females using the chi-square test for categorical variables and either the independent t-test or Mann–Whitney U test for continuous variables. To compare the clinical and laboratory data among the three groups (low, high, and extremely high HDL-C), we used the chi-square test for categorical variables and analysis of variance and post hoc Scheffe’s test or Kruskal–Wallis test for continuous variables.

With the sex-stratified three-group model, we compared the 10-year mortality between groups using the chi-square test. We performed unadjusted and adjusted Cox proportional hazard analyses for males and females using the HDL-C 40–90 mg/dL and 50–90 mg/dL as the references, respectively. We selected covariates for statistical adjustment from the essential parameters of the ASCVD risk calculator and modified them based on clinical judgment as previously described [4]. We drew frequency distribution bars of 10-year mortality in each HDL-C 10 mg/dL interval with a trend line using Microsoft Excel 2016 (Microsoft Co, Redmond, WA, USA). All other analyses were performed using SAS Enterprise Guide 7.1 software (SAS Institute Inc., Cary, NC, USA). *p* values (two-tailed) less than 0.05 were considered statistically significant.

## 3. Results

### 3.1. Baseline Characteristics

**Figure 2:**
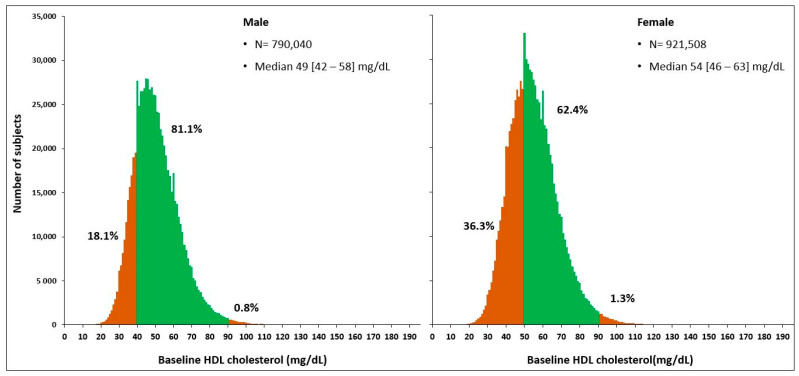
Distribution of the baseline HDL-cholesterol concentrations in subjects (≥40 years) with atherosclerotic cardiovascular diseases. Based on data from the 2010 Korean National Health Checkup, the subdivisions in each sex indicate the three-group model determined by HDL cholesterol levels.

The study included a total of 1,711,548 participants, out of which 790,040 (46.2%) were male and 921,508 (53.8%) were female. The average age of the participants was 61.4 years. The baseline characteristics of the study population are presented in Table 1. Hypertension and diabetes mellitus were more prevalent in males than in females (*p* < 0.0001). The baseline HDL-C level was significantly higher in females than in males (54 [46–63] mg/dL vs. 49 [42–58] mg/dL, *p* < 0.0001) (Figure 2). The LDL-C and Non-HDL-C levels were also higher in females, while the level of triglycerides was higher in males (*p* < 0.0001).

Of all the subjects, 27.9% had low levels of HDL-C, and only 1.1% had extremely high levels of HDL-C. Among males, 18.1% had low HDL-C levels, and 0.8% had extremely high levels of HDL-C. In females, 36.3% had low HDL-C levels, and 1.3% had extremely high HDL-C levels. Table 2 displays the clinical and laboratory characteristics of three HDL-C groups in each sex. In both sexes, fasting glucose, triglycerides, and Non-HDL-C were significantly higher in the low HDL-C group (all *p* < 0.0001), while LDL-C was significantly higher in the high HDL-C group (*p* < 0.001). In males, both systolic and diastolic BP were significantly higher in the group with extremely high HDL-C (*p* < 0.0001).

### 3.2. Sex-Stratified Three-Group Comparisons of 10-Year All-Cause Mortality

Over a 10-year period, 218,252 (12.8%) of the total cohort died. Males had a significantly higher 10-year mortality rate than females (16.2% vs. 9.8%; *p* < 0.001). Table 3 presents the 10-year mortality rates categorized by baseline HDL-C levels. For all subjects, the low HDL-C group demonstrated a higher 10-year mortality rate than the high or extremely high HDL-C groups (14.4% vs. 12.1% or 13.0%, *p* < 0.0001). The extremely high HDL-C group had a higher 10-year mortality rate than the high HDL-C group (*p* < 0.0001). Among males, the group with high HDL-C levels also had the best 10-year survival rate compared to other groups (*p* < 0.0001). However, the group with extremely high HDL-C levels showed a significantly higher 10-year mortality rate than the low HDL-C groups (21.6% vs. 19.4%, *p* < 0.0001). Among females, a significantly higher 10-year mortality rate was observed in the low HDL-C group (*p* < 0.001), but there was no difference between the high and extremely high HDL-C groups (8.4% vs. 8.4%, *p* = 0.94).

### 3.3. Hazard Ratios for 10-Year All-Cause Mortality

The sex-stratified Cox proportional hazard regression analysis results for 10-year all-cause mortality are in Table 4. For males, having low levels of HDL-C or extremely high levels of HDL-C is associated with increased 10-year mortality risks of 29.6% or 46.4%, respectively, compared to having high levels of HDL-C. The risk remained significantly higher at 18.3% and 35.9%, respectively, even after adjustment (*p* < 0.0001). For females, having low levels of HDL-C was associated with a significantly higher risk of mortality of 47.2% over the next 10 years compared to having high levels of HDL-C (*p* < 0.0001). However, females with extremely high levels of HDL-C had a similar risk to those with high levels of HDL-C (*p* = 0.85). Only after adjusting for the effects of covariates did the extremely high HDL-C group show a significant increase in risk of 9.5% compared to the high HDL-C group (*p* = 0.0041).

### 3.4. Distribution of 10-Year Mortality Stratified by Baseline HDL-C Levels

**Figure 3:**
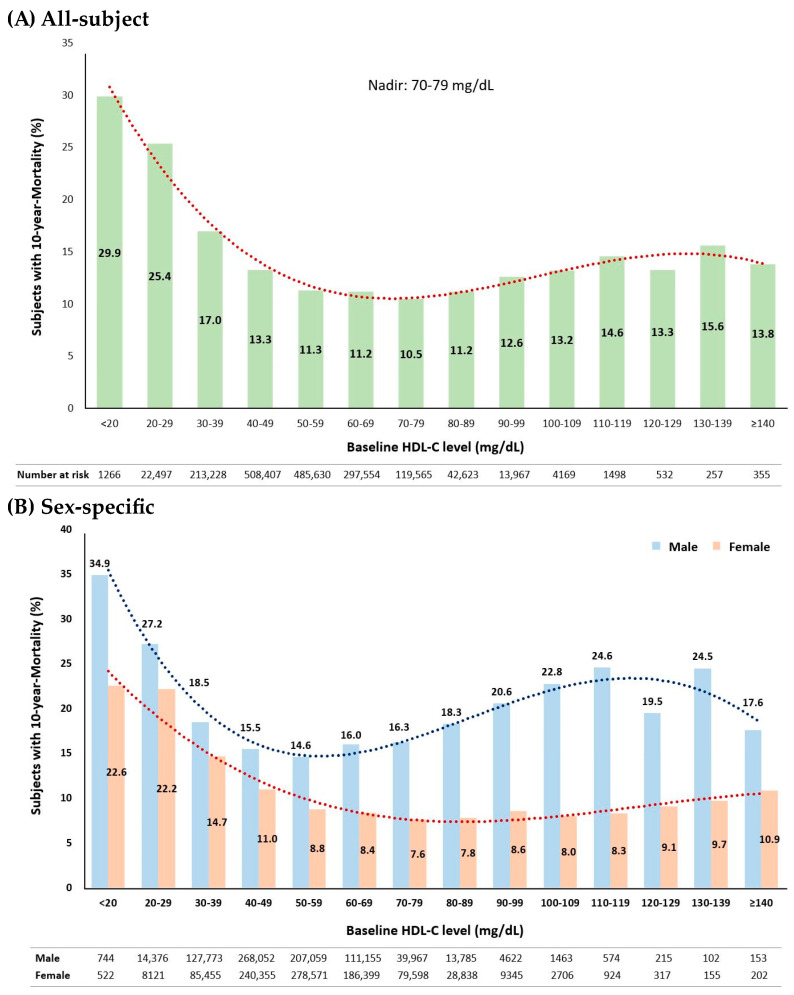
Percentage distribution bars of subjects with 10-year mortality by baseline HDL-C levels in 10 mg/dL intervals. (**A**) For all subjects (N = 1,711,548), a polynomial trend line (red dots) shows R^2^ of 0.975 (y = 0.0002x^4^ − 0.0507x^3^ + 1.3332x^2^ − 11.229x + 40.77). (**B**) For each sex (males, N = 790,040; females, N = 921,508), a slightly U-shaped pattern exists with different inflection points. Blue bars represent the percentage distribution of male subjects, with the nadir at 50–59 mg/dL; red bars for female subjects, with the nadir at 70–79 mg/dL. For males, a polynomial trendline (blue dots) fit R^2^ of 0.929 (y = 0.0016x^4^ − 0.1273x^3^ + 16.314x + 49.408); for females, a polynomial trendline (red dots) fit R^2^ of 0.951 (y = −0.0192x^3^ + 0.666x^2^ − 6.9935x + 30.529).

This study with over 1.7 million individuals revealed a slightly U-shaped curve relating baseline HDL-C levels to the 10-year mortality rate. The HDL-C range of 70–79 mg/dL exhibited the lowest mortality rate of 10.5% (Figure 3A). When analyzed by sex, the inflection point occurred earlier in males than in females (Figure 3B). In males, the nadir interval was 50–59 mg/dL with a 10-year mortality rate of 14.6%, while in females, the nadir interval was 70–79 mg/dL with a 10-year mortality rate of 7.6%.

## 4. Discussion

This study of a large clinical ASCVD population shows that there is a slightly U-shaped relationship between HDL-C levels and 10-year mortality in both sexes, with earlier inflection in males (nadir: 50–59 mg/dL in males, 70–79 mg/dL in females). The extremely high HDL-C level (>90 mg/dL) increased the risk of 10-year all-cause mortality by 35.9% and 9.5% compared with the reference HDL-C levels of 40–90 mg/dL in males and of 50–90 mg/dL in females, independent of age, BMI, LDL-C, triglycerides, hypertension, diabetes mellitus, smoking, and alcohol consumption.

So far, only two large-scale studies have investigated the association between HDL-C levels and all-cause mortality in clinical ASCVD subjects. Kaur et al. [9] conducted a study (N = 15,633, male 70%, mean age 65.8 years) in the Cleveland Clinic, enrolling subjects from 2005 to 2017, with follow-ups ending in 2018. They discovered a U-shaped correlation between HDL-C levels and overall mortality in patients who underwent percutaneous coronary intervention. The study found that the optimal HDL-C levels were 30–50 mg/dL for all subjects, but this U-shaped curve was not observed in females. Liu et al. [10] conducted a study on patients with coronary artery disease (N = 14,478, male 76%, mean age 62.1 years) recruited from the UK Biobank, with a median follow-up of 8.9 years. The study found that people with extremely high levels of HDL-C (>80 mg/dL) had a 96% greater risk of all-cause mortality compared to those with HDL-C levels ranging from 40–60 mg/dL. However, the study did not deeply explore the relationship between HDL-C levels and mortality by sex.

Our current study has a stronger basis than the two previous ASCVD studies as it involves a well-balanced and substantially larger population (N = 1,711,548, female 54%) and analyzes the association by sex. Additionally, all individuals were followed for a period of 10 years. Kaur et al. were unable to show a U-shaped curve in females, possibly reflecting sparse data for extremely high HDL-C levels in females, which is similar to Liu et al. [11,12]. The extremely high HDL-C levels are sex-specific and generally have higher ranges in females than males [1,2,4]. Ultimately, adjusting traditional risk factors, including age, our study successfully demonstrated the extremely high HDL-C levels (>90 mg/dL), showing increased 10-year mortality risk compared with the high HDL-C ranges as a reference value in both sexes (Table 4).

In this ASCVD cohort, we observed significant differences in baseline characteristics between males and females (Table 1). As expected, there were differences in age and serum creatinine levels, with females tending to have longer expected lifespans [13] and relatively less muscle mass [14]. Females had lower TG but higher TC, HDL-C, LDL-C, and Non-HDL-C than males. In the elderly, TC and LDL-C levels gradually decline, especially in males [15]. Our study included females with a mean age of 62, which presumably entails a substantial postmenopausal decrease in estrogen production and an increase in LDL-C levels [15,16].

The sex-stratified three-group model, categorized with HDL-C levels, may not be perfect, especially for females. We believe that the upper limits of the optimal HDL-C level should be adjusted to higher levels for females. However, the current three-group model still provides clinically meaningful information. After excluding the highest age subgroup (≥80 years), the extremely high HDL-C group consistently showed a higher 10-year mortality rate than the other HDL-C groups in all subjects and males (Table 3). When excluding the age ≥ 80 years, the female group with extremely high HDL-C also exhibited higher mortality rates than the high HDL-C group or at least a statistically equal risk to the low HDL-C group (Table 3). Therefore, we suggest that individuals with clinical ASCVD, aged between 40–79 years, and who have extremely high levels of HDL-C (≥90 mg/dL) have an increased risk of 10-year all-cause mortality compared to the optimal reference in their age subgroup.

Our study has found an unexpected, interesting result. We have observed that the level of LDL-C is significantly higher in the high HDL-C group, which is the group with the best survival rate, than in the other groups, irrespective of sex. Similar studies have been reported, such as the non-association of LDL-C levels with the residual risk of all-cause mortality in statin-treated patients [17] or an inverse relationship between LDL-C levels and the risk of all-cause mortality [18,19]. Therefore, other lipid biomarkers, such as HDL-C, might be more promising than LDL-C for 10-year survival prognoses.

It is not clear why having an extremely high level of HDL-C is connected to a higher risk of all-cause mortality. This association does not necessarily mean causation; it could indicate underlying contributing conditions. In some cases, conditions like inflammatory diseases, thyroid disorders, liver disorders, and acute coronary syndrome can cause HDL particles to become dysfunctional or reduce the cholesterol efflux capacity [20,21,22,23,24]. A similar U-shaped relationship was also observed between HDL-C levels and the risk of hip fracture in older adults [25]. Although the mechanism is unclear, in individuals with ASCVD, mostly those on LDL-C suppression for secondary prevention, HDL-C levels can be a prognostic marker of 10-year mortality prediction.

HDL-C is one of the essential factors in the pooled cohort ASCVD risk equation [26]. The current ACC/AHA-derived ASCVD risk calculator still considers HDL-C as a linear negative predictor in the range of 20–100 mg/dL [27]. Optimal HDL-C levels have a lower cut-off value of 40 mg/dL for men and 50 mg/dL for women [7], but there is no concern regarding the sex-specific upper optimal levels. Our study data emphasize the importance of sex-specific HDL-C reference values because, first, the distribution of HDL-C concentration is different between males and females (49 [42–58] mg/dL vs. 54 [46–63] mg/dL, *p* < 0.0001), and second, the frequency distribution bars for the 10-year mortality rate also showed sex-specific nadirs of 50–59 mg/dL in males and 70–79 mg/dL in females, with an early sharp inflection in males. Therefore, further studies are needed to determine the sex-specific optimal upper HDL-C levels for clinical practice.

It is necessary to recognize the limitations of this study. First, it was a retrospective cohort study. Second, this study only includes South Koreans; the findings are not necessarily generalizable. Third, we only examined the primary endpoint of all-cause mortality instead of major adverse CV events. We did this because the coding system cannot completely distinguish between new and underlying CV events. Additionally, the National Death Registry of Korea only provides dates of death without any information about the cause of death. Fourth, we analyzed the HDL-C levels at three-group or 10 mg/dL intervals instead of continuous analysis. For daily clinical use, it would be more practical to have a simple range for optimal HDL-C levels. Future studies should focus not only on HDL-C levels but also on the qualitative and functional assessment of HDL-C, including subclasses and particles [23,24,28,29].

## 5. Conclusions

Our study, with a large clinical ASCVD population, showed a slightly U-shaped correlation between HDL-C levels and a 10-year mortality rate with an earlier inflection in males. Males had a lower nadir (50–59 mg/dL) than females (70–79 mg/dL). HDL-C levels >90 mg/dL increased the risk of 10-year all-cause mortality by 35.9% in males and 9.5% in females compared to reference levels (40–90 mg/dL in males, 50–90 mg/dL in females).

## Figures and Tables

**Table 1:** Baseline characteristics.

	Total	Male	Female	*p* Value
Number of subjects, n (% of total cohort)	1,711,548 (100)	790,040 (46.2)	921,508 (53.8)	_
Age, years	61.4 ± 10.3	60.7 ± 10.3	62.1 ± 10.3	<0.0001
40–49 years, n (%)	227,898 (13.3)	122,244 (15.5)	105,654 (11.5)	<0.0001
50–59 years, n (%)	521,167 (30.5)	246,790 (31.2)	274,377 (29.3)	
60–69 years, n (%)	533,907 (31.2)	242,738 (30.7)	291,169 (31.6)	
70–79 years, n (%)	361,140 (21.1)	151,911 (19.2)	209,229 (22.7)	
≥80 years, n (%)	67,436 (3.9)	26,357 (3.3)	41,079 (4.5)	
BMI, kg/m^2^	24.47 ± 3.79	24.46 ± 2.97	24.48 ± 4.36	<0.0001
Obesity, n (%)	691,695 (40.4)	322,504 (40.8)	369,191 (40.1)	<0.0001
Abd. obesity, n (%)	540,209 (31.6)	247,495 (31.3)	292,714 (31.8)	<0.0001
Smoking, n (%)				
Never	1,164,024 (68.2)	278,817 (35.4)	885,207 (96.3)	<0.0001
Past	291,584 (17.1)	271,184 (35.7)	10,400 (1.1)	
Current	252,169 (14.8)	228,484 (29.0)	23,685 (2.6)	
Alcohol, n (%)				
None	1,163,813 (68.2)	355,762 (45.1)	808,051 (87.9)	<0.0001
1 time/week	205,152 (12.0)	138,401 (17.6)	66,751 (7.3)	
2 times/week	135,159 (7.9)	112,196 (14.2)	22,963 (2.5)	
≥3 times/week	203,297 (11.9)	181,860 (23.1)	21,437 (2.3)	
Systolic BP, mmHg	127.3 ± 15.7	128.0 ± 15.1	126.7 ± 16.1	<0.0001
Diastolic BP, mmHg	78.1 ± 10.0	78.9 ± 9.9	77.4 ± 10.0	<0.0001
Hypertension, n (%)	1,044,507 (61.0)	497,307 (63.0)	547,200 (59.4)	<0.0001
DM, n (%)	399,287 (23.3)	210,225 (26.6)	189,062 (20.5)	<0.0001
FBS, mg/dL	98 [89–110]	99 [90–114]	96 [88–107]	<0.0001
Blood test at baseline				
sCr, mg/dL	0.9 [0.8–1.0]	1.0 [0.9–1.1]	0.8 [0.7–0.9]	<0.0001
TC, mg/dL	194 [168–220]	187 [162–212]	199 [174–226]	<0.0001
LDL-C, mg/dL	112 [89–137]	107 [84–130]	117 [94–142]	<0.0001
HDL-C, mg/dL	51 [44–61]	49 [42–58]	54 [46–63]	<0.0001
Triglycerides, mg/dL	122 [86–175]	128 [90–186]	117 [84–166]	<0.0001
Non-HDL-C, mg/dL	140 [116–167]	136 [112–162]	144 [120–171]	<0.0001

Values are mean ± standard deviation, number (%), or median [interquartile range]. *p* value between males and females: chi-square test, independent t-test, or Mann–Whitney U test. Missing data are shown in Supplemental Data Table S1. Obesity, BMI > 25 kg/m^2^; Abd. obesity, waist circumference ≥90 cm in males, ≥85 cm in females. Abbreviations: HDL-C, high-density lipoprotein cholesterol; n, number; BMI, body mass index; Abd. obesity, abdominal obesity; BP, blood pressure; DM, diabetes mellitus; FBS, fasting blood sugar; sCr, serum creatinine; TC, total cholesterol; LDL-C, low-density lipoprotein cholesterol.

**Table 2:** Comparison of the three groups categorized with HDL-cholesterol levels in each sex.

Sex	Male				Female			
Groups	Low (N = 142,893)	High (N = 640,800)	Extremely High (N = 6347)	*p* Value	Low (N = 334,453)	High (N = 574,941)	Extremely High (N = 12,114)	*p* Value
Age, years	61.3 ±10.4	60.5 ±10.3	61.5 ±10.1	<0.0001 ^†^	63.7 ±10.2	61.3 ±10.2	59.7 ±10.4	<0.0001 *
40–49 years, n (%)	20,436 (14.3)	101,005 (15.8)	803 (12.7)	<0.0001 *	29,538 (8.8)	74,137 (12.9)	1979 (16.3)	<0.0001 *
50–59 years, n (%)	43,114 (30.2)	201,757 (31.5)	1919 (30.2)		86,503 (25.9)	183,488 (31.9)	4386 (36.2)	
60–69 years, n (%)	43,616 (30.5)	197,005 (30.7)	2117 (33.4)		110,614 (33.1)	177,331 (30.8)	3224 (26.6)	
70–79 years, n (%)	30,185 (21.1)	120,434 (18.8)	1292 (20.4)		88,328 (26.4)	118,803 (20.7)	2098 (17.3)	
≥80 years, n (%)	5542 (3.9)	20,599 (3.2)	216 (3.4)		19,470 (5.8)	21,182 (3.7)	427 (3.5)	
BMI, kg/m^2^	25.1 ±2.9	24.3 ±3.0	22.8 ±3.1	<0.0001 *	24.8 ±5.8	24.3 ±3.3	23.4 ±3.4	<0.0001 *
Obesity, n (%)	70,045 (49.1)	251,065 (39.2)	1394 (22.0)	<0.0001 *	148,502 (44.5)	217,241 (37.8)	3448 (28.5)	<0.0001 *
Abd. obesity, n (%)	56,497 (39.6)	189,853 (29.6)	1145 (18.0)	<0.0001 *	123,811 (37.1)	166,292 (28.9)	2611 (21.6)	<0.0001 *
Smoking, n (%)								
Never	49,534 (34.7)	227,058 (35.5)	2225 (35.1)	<0.0001 ^†^	320,843 (96.1)	552,874 (96.4)	11,490 (95.0)	<0.0001 *
Past	48,475 (34.0)	230,583 (36.1)	2126 (33.5)		3732 (1.1)	6476 (1.1)	192 (1.6)	
Current	44,583 (31.3)	181,912 (28.4)	1989 (31.4)		9496 (2.8)	14,081 (2.5)	408 (3.4)	
Alcohol, n (%)								
None	81,074 (56.9)	273,028 (42.7)	1660 (26.3)	<0.0001 *	305,653 (91.6)	493,298 (86.0)	9100 (75.5)	<0.0001 *
1 time/week	25,139 (17.6)	112,481 (17.6)	781 (12.4)		18,470 (5.5)	46,960 (8.2)	1321 (11.0)	
2 times/week	15,893 (11.2)	95,385 (14.9)	981 (14.5)		5133 (1.5)	17,073 (3.0)	757 (6.3)	
≥3 times/week	20,471 (14.4)	158,434 (24.8)	2955 (46.8)		4540 (1.4)	16,018 (2.8)	879 (7.3)	
Systolic BP, mmHg	127.5 ±15.1	128.1 ±15.1	130.9 ±16.3	<0.0001 *	127.5 ±16.1	126.3 ±16.1	126.3 ±16.3	<0.0001 ^‡^
Diastolic BP, mmHg	78.3 ±10.0	79.0 ±9.9	80.6 ±10.4	<0.0001 *	77.5 ±10.0	77.2 ±10.0	77.8 ±10.3	<0.0001 ^†^
Hypertension, n (%)	94,843 (66.4)	398,574 (62.2)	3890 (61.3)	<0.0001 ^‡^	215,857 (64.5)	325,151 (56.6)	6192 (51.1)	<0.0001 *
DM, n (%)	47,168 (33.0)	161,636 (25.2)	1421 (22.4)	<0.0001 *	84,362 (25.2)	102,899 (17.9)	1801 (14.9)	<0.0001 *
Blood test at baseline								
FBS, mg/dL	101 [91–118]	99 [90–113]	100 [90–113]	<0.0001 ‡	97 [89–110]	96 [88–106]	95 [87–105]	<0.0001 *
sCr, mg/dL	1.0 [0.9–1.2]	1.0 [0.9–1.1]	1.0 [0.9–1.1]	<0.0001 *	0.8 [0.7–0.9]	0.8 [0.7–0.9]	0.8 [0.7–0.9]	<0.0001 ^‡^
TC, mg/dL	174 [149–199]	189 [165–215]	212 [188–239]	<0.0001 *	191 [166–218]	203 [179–230]	224 [199–252]	<0.0001 *
LDL-C, mg/dL	102 [79–125]	108 [85–131]	91 [67–117]	<0.0001 *	116 [93–141]	118 [96–143]	106 [82–133]	<0.0001 *
HDL-C, mg/dL	35 [32–38]	52 [45–60]	97 [93–105]	<0.0001 *	43 [39–46]	60 [54–67]	97 [93–103]	<0.0001 *
Triglycerides, mg/dL	164 [115–237]	121 [86–175]	89 [64–129]	<0.0001 *	145 [104–202]	106 [77–145]	83 [61–116]	<0.0001 *
Non-HDL-C, mg/dL	139 [115–165]	135 [111–161]	111 [86–138]	<0.0001 *	149 [124–175]	142 [118–168]	125 [100–153]	<0.0001 *

Values are mean ± standard deviation, number (%), or median [interquartile range]. *p* values are from the analysis of variance procedure, Kruskal–Wallis test, or chi-square test in the three groups. * low vs. high or extremely high, high vs. extremely high; ^†^ high vs. low or extremely high; ^‡^ low vs. high or extremely high in post hoc analysis using Scheffe’s test or two-sample chi-square test. For abbreviations, see Table 1.

**Table 3:** Ten-year mortality categorized by baseline HDL-C levels in each sex.

All Subjects	Total (N = 1,711,548)	Low (N = 477,346)	High (N = 1,215,741)	Extremely High (N = 18,461)	*p* Value
Total, n (%)	218,252 (12.8)	68,615 (14.4)	147,243 (12.1)	2394 (13.0)	<0.0001 *
By age, n (% of each age subgroup)					
40–49 years	4379 (1.9)	1037 (2.1)	3248 (1.9)	94 (3.4)	<0.0001 *
50–59 years	19,237 (3.7)	4835 (3.7)	14,108 (3.7)	294 (4.7)	<0.0001 ^†^
60–69 years	53,495 (10.0)	15,091 (9.8)	37,774 (10.1)	630 (11.8)	<0.0001 *
70–79 years	99,699 (27.6)	32,118 (27.1)	66,577 (27.8)	1004 (29.6)	<0.0001 *
≥80 years	41,442 (61.5)	15,534 (62.1)	25,536 (61.1)	372 (57.9)	0.0068 ^†^
**Males**	**Total** **(N = 790,040)**	**Low** **(N = 142,893)**	**High** **(N = 640,800)**	**Extremely High (N = 6347)**	***p* value**
Male, n (%)	127,821 (16.2)	27,782 (19.4)	98666 (15.4)	1373 (21.6)	<0.0001 *
By age, n (% of each age subgroup)					
40–49 years, n (%)	3188 (2.6)	631 (3.1)	2498 (2.5)	59 (7.3)	<0.0001 *
50–59 years, n (%)	13,695 (5.5)	2824 (6.6)	10,671 (5.3)	200 (10.4)	<0.0001 *
60–69 years, n (%)	35,587 (14.7)	7532 (17.3)	27,651 (14.0)	404 (19.1)	<0.0001 *
70–79 years, n (%)	56,710 (37.3)	12,658 (41.9)	43,492 (36.1)	560 (43.3)	<0.0001 *
≥80 years, n (%)	18,641 (70.7)	4137 (74.6)	14,354 (69.7)	150 (69.4)	<0.0001 ^‡^
**Females**	**Total** **(N = 921,508)**	**Low** **(N = 334,453)**	**High** **(N = 574,941)**	**Extremely High (N = 12,114)**	***p* value**
Female, n (%)	90,431 (9.8)	40,833 (12.2)	48577 (8.4)	1021 (8.4)	<0.0001 ^‡^
By age, n (% of each age subgroup)					
40–49 years, n (%)	1191 (1.1)	406 (1.4)	750 (1.0)	35 (1.8)	<0.0001 *
50–59 years, n (%)	5542 (2.0)	2011 (2.3)	3437 (1.9)	94 (2.1)	<0.0001 ^§^
60–69 years, n (%)	17,908 (6.2)	7559 (6.8)	10,123 (5.7)	226 (7.0)	<0.0001 ^||^
70–79 years, n (%)	42,989 (20.5)	19,460 (22.0)	23,085 (19.4)	444 (21.2)	<0.0001 ^||^
≥80 years, n (%)	22,801 (55.5)	11,397 (58.5)	11,182 (52.8)	222 (52.0)	<0.0001 ^‡^

*p* values are from the chi-square test in the three groups. In the post hoc analyses, *p* < 0.05 in * low vs. high or extremely high, high vs. extremely high; ^†^ extremely high vs. low or high; ^‡^ low vs. high or extremely high; ^§^ low vs. high; ^||^ high vs. low or extremely high.

**Table 4:** Hazard ratios for 10-year mortality by sex.

Sex	Reference HDL-C	Group of HDL-C	Unadjusted HR (95% CI)	*p* Value	Adjusted HR * (95% CI)	*p* Value *
Male	40–90 mg/dL	Low	1.296 (1.279–1.314)	<0.0001	1.183 (1.166–1.199)	<0.0001
		Extremely high	1.464 (1.388–1.545)	<0.0001	1.359 (1.288–1.434)	<0.0001
Female	50–90 mg/dL	Low	1.472 (1.453–1.492)	<0.0001	1.153 (1.138–1.169)	<0.0001
		Extremely high	0.994 (0.934–1.053)	0.853	1.095 (1.029–1.167)	0.0041

* Adjusted for age, BMI, LDL-C, triglycerides, hypertension, diabetes mellitus, smoking, and alcohol consumption. Variables refer to Table 1. Abbreviations: HDL-C, high-density lipoprotein cholesterol; HR, hazard ratios; CI, confidence interval.

## Data Availability

All the data described in this study are available within the article or its Supplementary Materials.

## Supplementary Materials

The following supporting information can be downloaded at: https://www.mdpi.com/article/10.3390/metabo13121175/s1, Table S1. The missing numbers.
