# Supplementary material for: Sex-Specific Relationships between HDL-Cholesterol Levels and 10-Year Mortality in Individuals with Atherosclerotic Cardiovascular Disease: A Nationwide Cohort Study of South Koreans
Source: Metabolites. 2023 Nov 26;13(12):1175. doi: 10.3390/metabo13121175 (PMC10744622; doi:10.3390/metabo13121175)
Supplement: Supplementary file 1 [file metabolites-13-01175-s001.zip › metabolites-2729569-supplementary.pdf]

Table S1. The missing numbers

	Total (N = 1,711,548)	Low HDL-C (N = 477,346)	High HDL-C (N = 1,215,741)	Extremely high HDL-C (N = 18,461)
Body mass index, N (%)	1158 (0.07)	566 (0.12)	588 (0.05)	4 (0.02)
Male, N	411	134	276	1
Female, N	747	432	312	3
Waist circumference, N (%)	1185 (0.07)	553 (0.12)	628 (0.05)	4 (0.02)
Male, N	444	128	315	1
Female, N	741	425	313	3
Smoking, N (%)	3771 (0.22)	983 (0.21)	2757 (0.23)	31 (0.17)
Male, N	1555	301	1247	7
Female, N	2216	682	1510	24
Alcohol, N (%)	4127 (0.24)	973 (0.20)	3064 (0.25)	90 (0.49)
Male, N	1821	316	1472	33
Female, N	2306	657	1592	57
